# Supplementary material for: Pyruvate Dehydrogenase Kinase 1 inhibition mediated oxidative phosphorylation enhancement in cartilage promotes osteoarthritis progression
Source: BMC Musculoskelet Disord. 2023 Jul 20;24:597. doi: 10.1186/s12891-023-06585-6 (PMC10357736; doi:10.1186/s12891-023-06585-6)
Supplement: Supplementary file 1 — Additional file 1. [file 12891_2023_6585_MOESM1_ESM.zip › KEGG.Gsea.1653622432464/KEGG_STEROID_HORMONE_BIOSYNTHESIS.html]

Details for gene set KEGG\_STEROID\_HORMONE\_BIOSYNTHESIS[GSEA]

|  || Dataset | 55457\_collapsed\_to\_symbols.cls.cls #OA\_versus\_normal.cls.cls #OA\_versus\_normal\_repos |
| Phenotype | cls.cls#OA\_versus\_normal\_repos |
| Upregulated in class | OA |
| GeneSet | KEGG\_STEROID\_HORMONE\_BIOSYNTHESIS |
| Enrichment Score (ES) | 0.24511562 |
| Normalized Enrichment Score (NES) | 0.96332157 |
| Nominal p-value | 0.5012225 |
| FDR q-value | 0.69109434 |
| FWER p-Value | 1.0 |
Table: GSEA Results Summary

  

Fig 1: Enrichment plot: KEGG\_STEROID\_HORMONE\_BIOSYNTHESIS      
 Profile of the Running ES Score & Positions of GeneSet Members on the Rank Ordered List

  

| SYMBOL | TITLE | RANK IN GENE LIST | RANK METRIC SCORE | RUNNING ES | CORE ENRICHMENT || 1 | CYP11A1 | cytochrome P450 family 11 subfamily A member 1 [Source:HGNC Symbol;Acc:HGNC:2590] | 313 | 0.740 | 0.0300 | Yes |
| 2 | COMT | catechol-O-methyltransferase [Source:HGNC Symbol;Acc:HGNC:2228] | 335 | 0.732 | 0.0821 | Yes |
| 3 | HSD11B2 | hydroxysteroid 11-beta dehydrogenase 2 [Source:HGNC Symbol;Acc:HGNC:5209] | 539 | 0.645 | 0.1137 | Yes |
| 4 | UGT2B4 | UDP glucuronosyltransferase family 2 member B4 [Source:HGNC Symbol;Acc:HGNC:12553] | 566 | 0.634 | 0.1583 | Yes |
| 5 | HSD17B8 | hydroxysteroid 17-beta dehydrogenase 8 [Source:HGNC Symbol;Acc:HGNC:3554] | 704 | 0.593 | 0.1911 | Yes |
| 6 | HSD17B12 | hydroxysteroid 17-beta dehydrogenase 12 [Source:HGNC Symbol;Acc:HGNC:18646] | 795 | 0.570 | 0.2260 | Yes |
| 7 | STS | steroid sulfatase [Source:HGNC Symbol;Acc:HGNC:11425] | 1254 | 0.467 | 0.2247 | Yes |
| 8 | SRD5A1 | steroid 5 alpha-reductase 1 [Source:HGNC Symbol;Acc:HGNC:11284] | 1973 | 0.364 | 0.1955 | Yes |
| 9 | AKR1C3 | aldo-keto reductase family 1 member C3 [Source:HGNC Symbol;Acc:HGNC:386] | 1996 | 0.361 | 0.2204 | Yes |
| 10 | UGT2B17 | UDP glucuronosyltransferase family 2 member B17 [Source:HGNC Symbol;Acc:HGNC:12547] | 2050 | 0.356 | 0.2424 | Yes |
| 11 | SULT1E1 | sulfotransferase family 1E member 1 [Source:HGNC Symbol;Acc:HGNC:11377] | 2791 | 0.271 | 0.2047 | Yes |
| 12 | UGT2A3 | UDP glucuronosyltransferase family 2 member A3 [Source:HGNC Symbol;Acc:HGNC:28528] | 2854 | 0.265 | 0.2193 | Yes |
| 13 | HSD17B6 | hydroxysteroid 17-beta dehydrogenase 6 [Source:HGNC Symbol;Acc:HGNC:23316] | 2965 | 0.254 | 0.2294 | Yes |
| 14 | HSD11B1 | hydroxysteroid 11-beta dehydrogenase 1 [Source:HGNC Symbol;Acc:HGNC:5208] | 3000 | 0.250 | 0.2451 | Yes |
| 15 | UGT1A8 | UDP glucuronosyltransferase family 1 member A8 [Source:HGNC Symbol;Acc:HGNC:12540] | 3301 | 0.221 | 0.2380 | No |
| 16 | CYP7B1 | cytochrome P450 family 7 subfamily B member 1 [Source:HGNC Symbol;Acc:HGNC:2652] | 3584 | 0.195 | 0.2304 | No |
| 17 | HSD3B1 | "hydroxy-delta-5-steroid dehydrogenase, 3 beta- and steroid delta-isomerase 1 [Source:HGNC Symbol;Acc:HGNC:5217]" | 3695 | 0.186 | 0.2355 | No |
| 18 | HSD3B2 | "hydroxy-delta-5-steroid dehydrogenase, 3 beta- and steroid delta-isomerase 2 [Source:HGNC Symbol;Acc:HGNC:5218]" | 4424 | 0.119 | 0.1876 | No |
| 19 | CYP7A1 | cytochrome P450 family 7 subfamily A member 1 [Source:HGNC Symbol;Acc:HGNC:2651] | 4499 | 0.113 | 0.1902 | No |
| 20 | CYP3A43 | cytochrome P450 family 3 subfamily A member 43 [Source:HGNC Symbol;Acc:HGNC:17450] | 4698 | 0.096 | 0.1818 | No |
| 21 | UGT1A10 | UDP glucuronosyltransferase family 1 member A10 [Source:HGNC Symbol;Acc:HGNC:12531] | 4850 | 0.083 | 0.1762 | No |
| 22 | AKR1D1 | aldo-keto reductase family 1 member D1 [Source:HGNC Symbol;Acc:HGNC:388] | 5836 | 0.006 | 0.1000 | No |
| 23 | AKR1C4 | aldo-keto reductase family 1 member C4 [Source:HGNC Symbol;Acc:HGNC:387] | 5843 | 0.005 | 0.1000 | No |
| 24 | CYP1B1 | cytochrome P450 family 1 subfamily B member 1 [Source:HGNC Symbol;Acc:HGNC:2597] | 5925 | 0.000 | 0.0937 | No |
| 25 | CYP19A1 | cytochrome P450 family 19 subfamily A member 1 [Source:HGNC Symbol;Acc:HGNC:2594] | 6388 | -0.034 | 0.0603 | No |
| 26 | UGT2B11 | UDP glucuronosyltransferase family 2 member B11 [Source:HGNC Symbol;Acc:HGNC:12545] | 7270 | -0.100 | -0.0009 | No |
| 27 | SRD5A3 | steroid 5 alpha-reductase 3 [Source:HGNC Symbol;Acc:HGNC:25812] | 7325 | -0.104 | 0.0026 | No |
| 28 | HSD17B1 | hydroxysteroid 17-beta dehydrogenase 1 [Source:HGNC Symbol;Acc:HGNC:5210] | 7940 | -0.150 | -0.0342 | No |
| 29 | CYP1A1 | cytochrome P450 family 1 subfamily A member 1 [Source:HGNC Symbol;Acc:HGNC:2595] | 8195 | -0.171 | -0.0414 | No |
| 30 | AKR1C1 | aldo-keto reductase family 1 member C1 [Source:HGNC Symbol;Acc:HGNC:384] | 8198 | -0.171 | -0.0290 | No |
| 31 | CYP3A5 | cytochrome P450 family 3 subfamily A member 5 [Source:HGNC Symbol;Acc:HGNC:2638] | 8272 | -0.177 | -0.0217 | No |
| 32 | CYP11B1 | cytochrome P450 family 11 subfamily B member 1 [Source:HGNC Symbol;Acc:HGNC:2591] | 8450 | -0.190 | -0.0215 | No |
| 33 | CYP3A7 | cytochrome P450 family 3 subfamily A member 7 [Source:HGNC Symbol;Acc:HGNC:2640] | 9024 | -0.234 | -0.0489 | No |
| 34 | UGT2A1 | UDP glucuronosyltransferase family 2 member A1 complex locus [Source:HGNC Symbol;Acc:HGNC:12542] | 9451 | -0.269 | -0.0622 | No |
| 35 | CYP21A2 | cytochrome P450 family 21 subfamily A member 2 [Source:HGNC Symbol;Acc:HGNC:2600] | 9493 | -0.273 | -0.0453 | No |
| 36 | SULT2B1 | sulfotransferase family 2B member 1 [Source:HGNC Symbol;Acc:HGNC:11459] | 9696 | -0.291 | -0.0397 | No |
| 37 | CYP11B2 | cytochrome P450 family 11 subfamily B member 2 [Source:HGNC Symbol;Acc:HGNC:2592] | 9951 | -0.316 | -0.0362 | No |
| 38 | SRD5A2 | steroid 5 alpha-reductase 2 [Source:HGNC Symbol;Acc:HGNC:11285] | 10149 | -0.336 | -0.0269 | No |
| 39 | CYP3A4 | cytochrome P450 family 3 subfamily A member 4 [Source:HGNC Symbol;Acc:HGNC:2637] | 10193 | -0.340 | -0.0053 | No |
| 40 | HSD17B2 | hydroxysteroid 17-beta dehydrogenase 2 [Source:HGNC Symbol;Acc:HGNC:5211] | 11148 | -0.451 | -0.0464 | No |
| 41 | HSD17B7 | hydroxysteroid 17-beta dehydrogenase 7 [Source:HGNC Symbol;Acc:HGNC:5215] | 11723 | -0.537 | -0.0516 | No |
| 42 | CYP17A1 | cytochrome P450 family 17 subfamily A member 1 [Source:HGNC Symbol;Acc:HGNC:2593] | 12154 | -0.632 | -0.0386 | No |
| 43 | UGT2B15 | UDP glucuronosyltransferase family 2 member B15 [Source:HGNC Symbol;Acc:HGNC:12546] | 12225 | -0.652 | 0.0038 | No |
| 44 | HSD17B3 | hydroxysteroid 17-beta dehydrogenase 3 [Source:HGNC Symbol;Acc:HGNC:5212] | 12253 | -0.662 | 0.0503 | No |
Table: GSEA details [plain text format]

  

Fig 2: KEGG\_STEROID\_HORMONE\_BIOSYNTHESIS      
 Blue-Pink O' Gram in the Space of the Analyzed GeneSet

  

Fig 3: KEGG\_STEROID\_HORMONE\_BIOSYNTHESIS: Random ES distribution      
 Gene set null distribution of ES for **KEGG\_STEROID\_HORMONE\_BIOSYNTHESIS**
